# Structure-Functional Basis of Ion Transport in Sodium–Calcium Exchanger (NCX) Proteins

**DOI:** 10.3390/ijms17111949

**Published:** 2016-11-22

**Authors:** Moshe Giladi, Reut Shor, Michal Lisnyansky, Daniel Khananshvili

**Affiliations:** 1 Department of Physiology and Pharmacology, Sackler Faculty of Medicine, Tel-Aviv University, Tel-Aviv 699780, Israel; reutshor@gmail.com (R.S.); michallis505@gmail.com (M.L.); 2 Tel-Aviv Sourasky Medical Center, Tel-Aviv 6423906, Israel

**Keywords:** alternating access, NCX (sodium–calcium exchanger), antiporter, transporter, occlusion, catalysis, selectivity, HDX-MS (hydrogen–deuterium exchange mass-spectrometry)

## Abstract

The membrane-bound sodium–calcium exchanger (NCX) proteins shape Ca^2+^ homeostasis in many cell types, thus participating in a wide range of physiological and pathological processes. Determination of the crystal structure of an archaeal NCX (NCX_Mj) paved the way for a thorough and systematic investigation of ion transport mechanisms in NCX proteins. Here, we review the data gathered from the X-ray crystallography, molecular dynamics simulations, hydrogen–deuterium exchange mass-spectrometry (HDX-MS), and ion-flux analyses of mutants. Strikingly, the apo NCX_Mj protein exhibits characteristic patterns in the local backbone dynamics at particular helix segments, thereby possessing characteristic HDX profiles, suggesting structure-dynamic preorganization (geometric arrangements of catalytic residues before the transition state) of conserved α_1_ and α_2_ repeats at ion-coordinating residues involved in transport activities. Moreover, dynamic preorganization of local structural entities in the apo protein predefines the status of ion-occlusion and transition states, even though Na^+^ or Ca^2+^ binding modifies the preceding backbone dynamics nearby functionally important residues. Future challenges include resolving the structural-dynamic determinants governing the ion selectivity, functional asymmetry and ion-induced alternating access. Taking into account the structural similarities of NCX_Mj with the other proteins belonging to the Ca^2+^/cation exchanger superfamily, the recent findings can significantly improve our understanding of ion transport mechanisms in NCX and similar proteins.

## 1. Introduction

A cascade of biological events at the cellular, organ, and systemic levels requires the direct or indirect contribution of distinct cations, such as H^+^, Na^+^, K^+^, Ca^2+^, and Mg^2+^, where protein structures can somehow manage the selective recognition of specific cations to couple the mechanical, catalytic, and transport activities possessed by a given protein [1,2,3]. Physiological concentrations of important cations in the extracellular, cytosolic, and subcellular (mitochondria, nuclei, and others) compartments are tightly controlled by membrane-intercalated proteins, channels, transporters and pumps, which can dynamically generate, retain, and modify ion homeostasis in time and space in accordance with the physiological demands of a given cell type [4,5,6]. However, identifying and quantifying the key conformational transitions governing the structure–functional specificity of ion-transporting proteins remains difficult, due to the limited capacities of current technologies for direct experimental measurement of the functionally relevant dynamic transitions. Although advanced computational approaches contributed significantly to a better understanding of the underlying mechanisms by providing a virtual route to complement the missing information, the rational integration of experimental data and theoretical calculations toward meaningful conclusions of fundamental significance remains challenging [1,2,3,7,8].

The alternating access mechanism in secondary-active transport systems refers to an alternative exposure of ligand binding domains at opposite sides of the membrane, while adopting at least two major conformational states, assigned as the inward-facing and outward-facing states [9,10,11]. Consistent with this general concept, recent crystallographic studies revealed the outward-facing, inward-facing, and occluded states in many transporters [12,13,14]. Despite these spectacular achievements in the structural biology of transporter proteins, the dynamic landscapes and functional assignments for the observed conformational states are still poorly understood. A thorough understanding of ligand-coupled alternating-access mechanisms remains challenging mainly because the dynamic features of transient intermediates throughout the transport cycle are incompletely resolved even for proteins with known crystal structures [9,10,11,12,13,14]. This is especially true for antiporter systems (like the sodium–calcium exchanger, NCX), where ligand interactions with proteins is “mandatory” (in contrast with e.g., uniporter systems) for alternating between the outward-facing and inward-facing states [7,8,9,10,11].

Although the structure-dynamic determinants of ion transport mechanisms were relatively well studied in ion channels [1,2,3], recent breakthroughs in the structural biology of ion transporters and pumps provided novel insights and conceptual frameworks for the systematic investigation of ion selectivity and transport mechanisms in these proteins [12,13,14]. Nevertheless, we are only at the beginning in understanding the details and mechanistic relevance of the conformational dynamics associated with alternating access, where applying new biophysical approaches in combination with computational sciences is required for making further progress. This review describes some new approaches that have been successfully applied for NCX proteins and that can be used for advanced investigation of many other transporters.

Over the last few years, major breakthroughs in our understanding of ion transport and selectivity by NCX have been made using diverse structural approaches (X-ray crystallography, molecular dynamics (MD), hydrogen–deuterium exchanger mass-spectrometry (HDX-MS), and functional (ion-flux) assays). The aim of this review is to summarize current understanding of NCX ion transport and selectivity at the molecular level.

## 2. Calcium Homeostasis and Physiological Relevance

Ca^2+^ is the most important and versatile secondary messenger [5,6], participating in a wide variety of cellular processes (e.g., hormone and neurotransmitter release, cardiac and skeletal muscle contraction–relaxation, apoptosis, and many other functions) in nearly all cell types. The special coordination chemistry of Ca^2+^ [15] allows it to readily bind to many different molecules; therefore, its resting free intracellular concentration has to be maintained at very low levels (~100 nM); uncontrolled Ca^2+^ rises in time and space may lead not only to unwanted pathophysiological processes but even to cell death. Thus, cellular Ca^2+^ concentrations require tight temporal and spatial regulation.

Regulation of intracellular Ca^2+^ levels involves a multitude of proteins, including various membrane proteins [5,6,16,17]. In many cell types, the Ca^2+^ extrusion from the cytosol is available mainly via the Ca^2+^-ATPase and the Na^+^/Ca^2+^ exchanger (NCX) proteins, and, thereby, these systems play a critical role in maintaining, handling and modifying the dynamic changes in the cellular Ca^2+^ levels [5,6,16,17]. The partial contribution of each of these transport systems differs among different cell types, which is pretty much dictated by physiological requirements of a given cell type [5,16,17]. Under most physiological conditions, NCX utilizes the electrochemical gradient for downhill movement of 3Na^+^ into the cell to mediate the uphill extrusion of 1Ca^2+^ from the cell [18], where during the transport cycle, the Na^+^ and Ca^2+^ ions are transported in separate steps through the NCX protein [19]. NCX proteins catalyze the exchange of one Ca^2+^ ion with three Na^+^ ions, where the directionality of the Ca^2+^-efflux (forward) or Ca^2+^-influx (reverse) modes depends on ionic concentrations and the membrane potential in a given cell type [16,17,20].

The gene family of mammalian NCX proteins consists of three gene isoforms (NCX1-3) which generate at least 17 splice variants and are expressed in a tissue-specific manner [20,21]. NCX proteins play a key role in regulating the Ca^2+^ homeostasis in many cell types, and, thus, modulate the cardiac rate and contractile force, blood pressure, kidney Ca^2+^ reabsorption, neurotransmitter and hormones secretion, apoptosis, cellular proliferation, and ATP synthesis rates in mitochondria, among other roles [16,17,20,21]. Usually, the reverse mode of NCX becomes predominant in pathological settings, which can significantly alter Ca^2+^ homeostasis with output affecting numerous Ca^2+^-dependent events that take place at the cellular or systemic levels. Since the protein expression levels and/or regulation of NCX isoform/variants are disease related in many pathological states (e.g., heart failure, cardiac arrhythmia, and cerebral ischemia), the selective pharmacological targeting of tissue-specific NCX variants could be beneficial, although this remains challenging [16,17].

## 3. X-ray Structure of Archaeal NCX Reveals the Architecture of the Ion Transport Domain

It has long been recognized that NCX might operate via an alternating-access mechanism, where the protein undergoes outward-facing (extracellular) and inward-facing (intracellular) conformational transitions upon Na^+^ or Ca^2+^ binding [19,22,23]. This conclusion has been drawn from kinetic analyses of ion-flux and electophysiological assays demonstrating that NCX translocates 3Na^+^ and 1Ca^2+^ in sequential steps (i.e., the ping-pong mechanism). However, only recently has a major advancement in understanding the structural basis for this mechanism been achieved by solving the crystal structure of NCX_Mj from the arachaeabacterium *Methanococcus jannaschii* [24]. In combination with known crystal structures, specially designed MD simulations and extended kinetic analyses of ion-fluxes in mutants revealed that the simultaneous occupation of 3Na^+^ and 1Ca^2+^ is thermodynamically forbidden [25,26,27]. Similarly to mammalian NCX, the NCX_Mj protein also translocates Na^+^ and Ca^2+^ with a stoichiometry of 3:1, although in contrast with NCX_Mj, the three mammalian genes NCX1-3 contain a large cytosolic regulatory loop (~520 aa) between helices 5 and 6 [28,29,30]. Thus, NCX_Mj is an ideal system for providing fundamental details regarding the ion-transport mechanisms in the NCX family.

**Figure 1:**
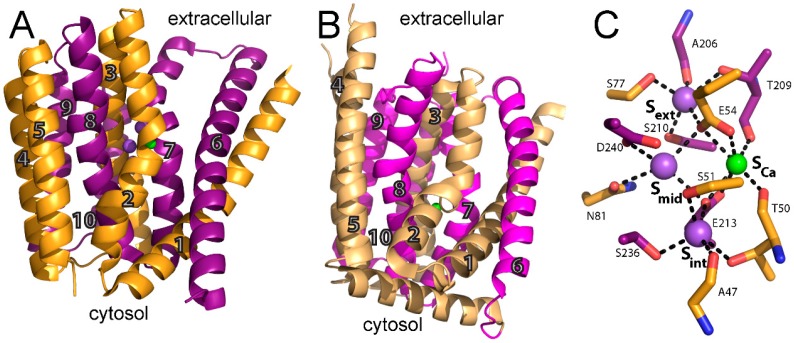
Structure of NCX_Mj (*Methanococcus jannaschii* sodium–calcium exchanger). (**A**) crystal structure of outward-facing NCX_Mj (PDB 3V5U) in cartoon representation. Helices 1–5 (TM1–5) are **orange** and helices 6–10 (TM6–10) are **purple**. **Purple** and **green** spheres represent Na^+^ and Ca^2+^ ions, respectively; (**B**) crystal structure of inward-facing VCX1 (PDB 4K1C) in cartoon representation. Helices 1–5 (TM1–5) are **light orange** and helices 6–10 (TM6–10) are **magenta**. **Green** sphere represents Ca^2+^; and (**C**) ion coordination as initially suggested by the crystal structure of NCX_Mj (PDB 3V5U). Ion coordinating residues are shown as sticks. **Purple** and **green** spheres represent Na^+^ and Ca^2+^ ions, respectively.

Notably, NCX_Mj was crystallized in the outward-facing occluded state in the presence of both Na^+^ and Ca^2+^ [24,27]. This structure reveals 10 transmembrane helices (TM) arranged in two pseudosymmetrical halves (TM1-5 and TM6-10) (Figure 1A). It was later shown that this topology also applies for mammalian NCX, again underscoring the utility of NCX_Mj as a model for the ion-transport domain [31]. TM1 and TM6 form the gating bundle, and the sliding of this two-helix cluster was suggested to be a major conformational change associated with the alternating-access during the transport cycle turnover [24]. This hypothesis is strongly supported by the X-ray structures of the H^+^/Ca^2+^ (CAX) exchangers that were solved after NCX_Mj [32,33,34], although the dynamic aspects of underlying transitions remain unresolved. Interestingly, to date, all H^+^/Ca^2+^ exchangers have crystallized in the inward-facing conformation. In the eukaryotic vacuolar H^+^/Ca^2+^ exchanger, VCX1, TM1 and TM6 are translated diagonally towards the vacuole (matching the extracellular side in NCX_Mj) by ~16 and ~13 Å at either end (Figure 1B) compared with the outward-facing state. This movement allows motion of the two pseudosymmetrical halves of the ion-transport core and closes the vacuole-facing entry passage of the ion-binding pocket, thus allowing conformational transitions associated with alternating access. A similar inward-facing state has been observed in an archaeal H^+^/Ca^2+^ exchanger from *Archaeoglobus fulgidus* [33].

In NCX_Mj and CAX proteins, TM2-5 and TM6-10 form the tightly packed core domain, where TM2-3 and TM7-8 contain signature α_1_ and α_2_-repeats, which are present in all members of the Ca^2+^/cation exchanger superfamily and are responsible for ion recognition and translocation. The α-repeats include twelve ion-coordinating residues (four in TM2 and TM7, and two in TM3 and TM8), forming four binding sites termed S_int_, S_mid_, S_ext_, and S_Ca_ in a diamond-shaped configuration. From the crystal structure, it is reasonable to assume that the S_ext_ and S_int_ sites are highly selective to Na^+^, whereas the S_mid_ and S_Ca_ sites do not exhibit any preferential ion selectivity. It was originally proposed that 3Na^+^ bind to S_int_, S_mid_, and S_ext_, or 1Ca^2+^ binds to S_Ca_ (Figure 1C). The proximity and ligand-sharing features of the binding sites were assumed to lead to an antagonist effect between Na^+^ and Ca^2+^. However, this interpretation of the ion binding sites was challenged by molecular dynamics and functional assays and was eventually revised using new X-ray structures (see below).

## 4. Structure-Functional Assignments of Four Binding Sites in NCX_Mj

As mentioned above, the original interpretation of the crystallographic data was that 3Na^+^ bind to S_int_, S_mid_, and S_ext_, whereas 1Ca^2+^ binds to S_Ca_ (Figure 1C). S_int_ and S_ext_ are both surrounded by five coordinating oxygens (A47, T50, S51, E213, and S236 in S_int_, A206, T209, S210, E54, and S77 in S_ext_) with features matching known Na^+^ binding sites. According to the original interpretation, the S_mid_ site is surrounded by only four coordinating oxygens (E54, N81, E213, and D240) and is not optimal for the binding of either Ca^2+^ or Na^+^ [24]. It was suggested that S_mid_ probably binds Na^+^ only at high concentrations, whereas S_Ca_ is surrounded by six coordinating oxygens (T50, E54, T209, and E213) consistent with ion-coordinating features of a Ca^2+^ binding site [24].

To resolve the uncertainty regarding the specificity and occupancy of the binding sites, especially S_mid_, molecular dynamics simulations were performed to systematically assess the different possible ion configurations [25]. This systematic analysis suggested that 3Na^+^ occupy S_int_, S_Ca_, and S_ext_ or that 1Ca^2+^ occupies S_Ca_, whereas in the Na^+^-bound state, D240 is protonated and a water molecule occupies S_mid_ [25]. This interpretation was verified experimentally by ion-flux assays of the D240N mutant, mimicking a constitutively protonated D240 (able to coordinate a water molecule) and exhibiting accelerated Na^+^/Ca^2+^ and Ca^2+^/Ca^2+^ exchange rates [25]. These data suggest that the deprotonation of D240 is not required for transport catalysis in NCX_Mj, even though transient deprotonation might occur initially during ion recognition. Therefore, D240 is protonated in both the Ca^2+^ and Na^+^ translocation steps, whereas, in the ground state, both ions do not bind to S_mid_. This interpretation is further supported by the lack of an effect of the N81A mutation either on the Na^+^/Ca^2+^ or Ca^2+^/Ca^2+^ exchange rates (notably, N81 is the only residue that exclusively belongs to the S_mid_ site, whereas the three other residues at S_mid_, E54, E213, and D240 participate in ion-coordination at other sites as well) [26]. Notably, kinetic analyses suggest that D240 may contribute to the transition state stabilization of ion-bound species, even though neither Ca^2+^ nor Na^+^ occupies the S_mid_ site in the ground state [26]. The protonation of D240 during the transport cycle also provides a simple explanation for the “confusing” observation that the conserved residue at position 240 in known prokaryotic and eukaryotic Na^+^/Ca^2+^ exchangers is an asparagine, and not an aspartate. Notably, the aspartate at 240 may account (at least partially) for the low turnover rates of the ion-exchange cycle of NCX_Mj [35], since the eukaryotic NCX orthologs with asparagine at 240 exhibit much higher turnover rates, which may have physiological significance for rapid extrusion of cytosolic Ca^2+^ in excitable tissues [16,17,35]. 

**Figure 2:**
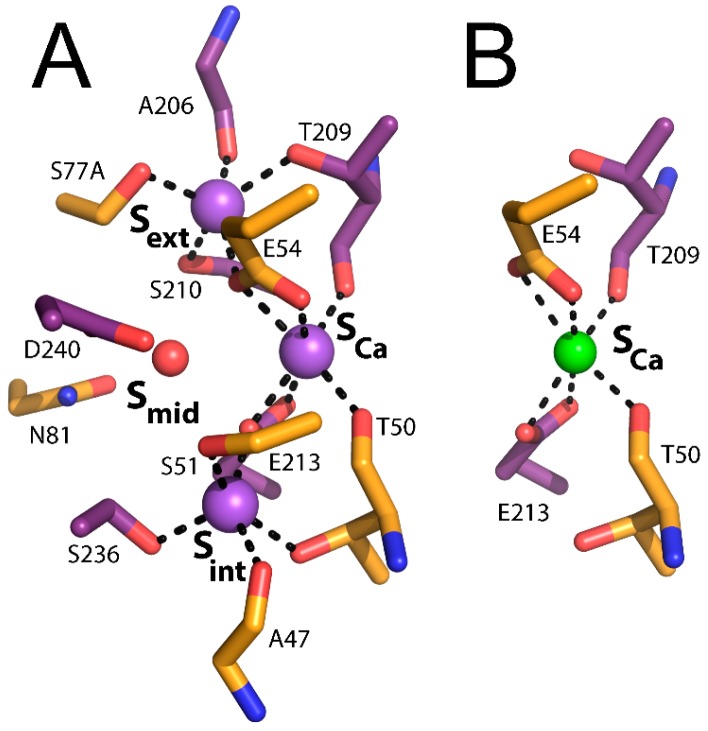
Ion binding sites of NCX_Mj. (**A**) Na^+^ binding sites (PDB 5HXE); (**B**) Ca^2+^ binding site (PDB 3V5U). Ion coordinating residues are presented as sticks. **Purple** and **green** spheres represent Na^+^ and Ca^2+^ ions, respectively.

Recently, a thorough structural analysis of ion recognition in the outward-facing state was performed by soaking NCX_Mj crystals in solutions with different ionic compositions [27]. These studies unequivocally confirm the model derived from the molecular dynamics and the ion-flux assays mentioned above. That is, 3Na^+^ bind to S_int_, S_Ca_, and S_ext_ and a water molecule occupies S_mid_, whereas Ca^2+^ binds to S_Ca_ (Figure 2A,B). Intriguingly, the 3Na^+^-bound conformation represents an outward-facing occluded state, whereas the Ca^2+^-bound conformation matches an outward-facing partially open state (see below).

## 5. Ion Transport Features of NCX_Mj

**Figure 3:**
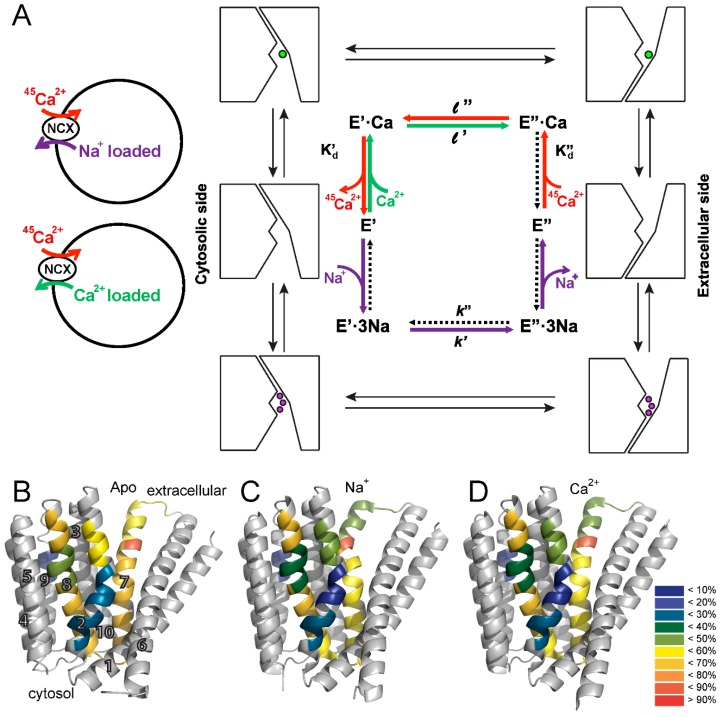
Ion transport cycle and NCX backbone dynamics. (**A**) schematic representation of the ion-flux assay for the Na^+^/^45^Ca^2+^ exchange or the Ca^2+^/^45^Ca^2+^ exchange and the ping-pong mechanism describing the exchange reactions. The **red**, **green**, and **purple** arrows represent Ca^2+^-entry, Ca^2+^-exit, and Na^+^-exit steps of the transport cycle, respectively. The dotted arrows represent the reactions of the transport cycle, which negligibly contribute to the observed ion-exchange reactions under the given experimental conditions. The K’_d_ and K”_d_ values represent the dissociation constants for Ca^2+^ binding to NCX_Mj at the cytosolic and extracellular sides, respectively; and (**B**–**D**) the heat map after a 1200 s exchange is overlaid on the crystal structure of NCX_Mj for the apo (PDB 5HXH, **B**), Na^+^-bound (PDB 5HXE, **C**), and Ca^2+^-bound (PDB 5HXR, **D**) forms. The color key indicates the HDX level. The numbers indicate the transmembrane helix number.

The structural information gained from the X-ray studies of NCX_Mj makes this protein an ideal system for studying ion transport by NCX proteins, since NCX_Mj lacks regulatory domains, while exhibiting striking similarities with mammalian NCXs in ion transport machinery [24,25,26,27,35]. The Khananshvili laboratory has extensively studied the functional properties of NCX_Mj by using ^45^Ca^2+^-uptake assays in *Escherichia coli*-derived vesicles containing overexpressed NCX_Mj protein and its mutants (Figure 3A). In this experimental system, the exchanger molecules are uniformly oriented in the isolated preparations of membrane vesicles, thereby allowing one to assess the cytosolic and extracellular K_m_ values for Ca^2+^ by varying intravesicular and extravesicular [Ca^2+^], respectively [25,26,35].

The functional analysis of NCX_Mj revealed a slow turnover rate of *k*_cat_ ~ 0.5 s^−1^, 10^3^–10^4^-fold slower than the turnover rate of mammalian NCX [16,17,35]. NCX_Mj displays functional asymmetry in Ca^2+^ transport, with an intrinsic equilibrium constant (K_int_) of ~0.15. K_int_ is calculated as the ratio between the cytosolic and extracellular K_m_ [26,35]. Since Ca^2+^ binds to the same site from both sides of the membrane, the difference in K_m_ reflects different rate constants (*l*’, *l*″) related to the intrinsic equilibrium of all species involved in alternating access (Figure 3). That is, Ca^2+^ movement from the cytosol to the extracellular side is ~seven-fold faster than in the opposite direction. A similar observation has also been made for mammalian NCX, despite the large difference in the turnover rate [17,35,36]. The intrinsic asymmetry of bidirectional ion movements in NCX has functional relevance, since under physiological conditions, NCX extrudes Ca^2+^ from the cytosol to the extracellular side [16,17,20,21].

To resolve the basis for the functional asymmetry of NCX_Mj, HDX-MS was performed on apo, Ca^2+^-, and Na^+^-bound NCX_Mj to detect differences in the backbone dynamics between pseudo-symmetric regions and the effect of ion binding on such asymmetry, if present [26]. HDX-MS measures the exchange of backbone amide hydrogen with deuterium in solvent, where the measured HDX is related to the solvent’s accessibility and the backbone folding/unfolding dynamics [37,38,39]. This analysis yielded several important insights. First, pseudo-symmetric regions in the ion binding pocket display markedly different HDX dynamics in the apo form of the protein (Figure 3B). That is, NCX_Mj displays structural asymmetry in the absence of ligand binding, emphasizing the fact that NCX_Mj is inherently asymmetrical to fulfill physiological requirements. Second, the differences in HDX reflect the preference for the outward-facing state, with regions exposed to the extracellular side taking up more deuterium compared with regions exposed to the cytosolic side during alternating access. Third, the presence of Na^+^ (Figure 3C) and Ca^2+^ (Figure 3D) results in subtle but specifically reduced HDX at the ion binding sites (see below), while maintaining a structural asymmetry that preferentially adopts the outward-facing state.

Despite continuous debates on specific contributions of physicochemical forces to catalytic preorganization and reorganization in biological catalyzators, the definition of catalytic preorganization remains to be fundamental for investigating the structure-based catalytic mechanisms [7,8]. In general, the catalytic preorganization refers to geometric arrangements of “catalytic residues” before the transition state is achieved. Typically, the structure-dynamic events associated with catalytic preorganization last for 10^−3^–10^−6^ s. The catalytic reorganization refers to catalytic rearrangement of side-chains in the transition state, which occurs in a much shorter time than the catalytic preorganization [7,8]. A structure-based mutational analysis of NCX_Mj was performed to assess the contribution of symmetry-related pair residues to ion transport. Interestingly, mutating the ion-coordinating residues led to a dramatic decrease in the turnover rate (*k*_cat_) but had a very minor effect on the intrinsic equilibrium (K_int_) [26,35]. These kinetic studies, in conjunction with HDX-MS analyses, have identified the ion-coordinating catalytic residues contributing to the stabilization of the Ca^2+^-bound transition state. Interestingly, the local backbone dynamics are much more constrained (less flexible) at S51 and E54 (TM2C) than at T209 (TM7B), E213 (TM7C), and D240 (TM8A), thereby suggesting a specific rearrangement (preorganization) of “catalytic side-chains” toward achieving the transition state. In contrast with the “catalytic residues”, mutations of distinct residues located at the entries of the ion-binding pocket or at the short loop between TM5 and TM6 led to dramatic changes in K_int_ (without significant alterations in *k*_cat_), thereby representing the stabilization of the cytosolic-facing state [26,35]. These results are intriguing, implying that structural and functional asymmetry are controlled separately from ion-transport catalysis. In light of the present considerations, it was concluded that the ion binding sites of NCX_Mj are asymmetrically preorganized in the apo state, followed by “catalytic” reorganization upon ligand binding, where the subsequent interactions with residues adjacent to the ion binding sites allow ion-coupled alternating access. How this coupling mechanism actually takes place in the framework of the “sliding” movement of the gating bundle (the TM1/TM6 cluster) [24] remains unclear, but one may posit that small conformational changes associated with “compression” of the ion-binding pocket upon the ion binding move the gating bundle toward the alternating access transition.

## 6. Ion-Bound Species and Alternating Access in NCX_Mj

**Figure 4:**
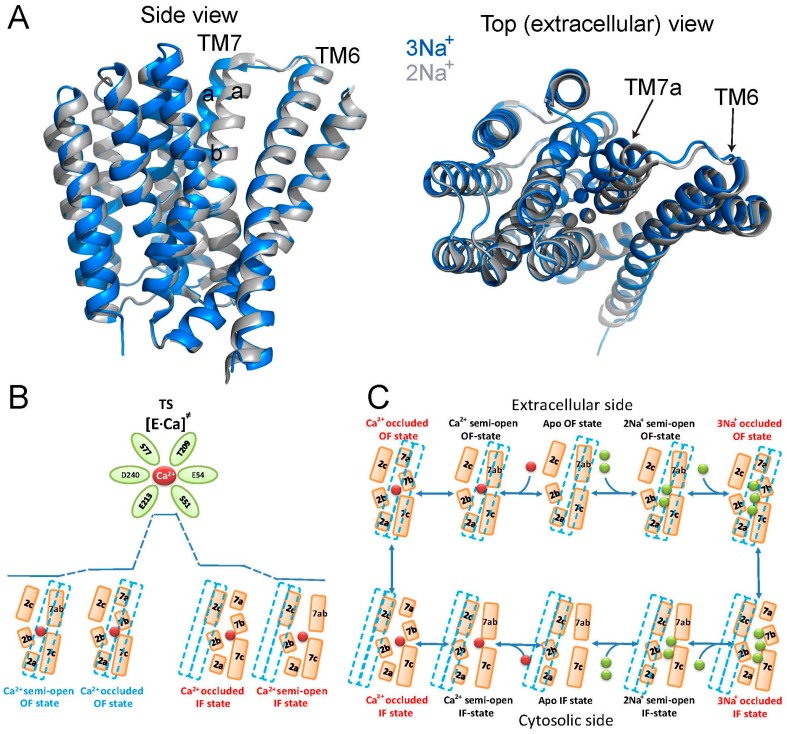
Ligand-induced alternating access in NCX_Mj. (**A**) the crystal structures of NCX_Mj bound to 3Na^+^ ions (**blue**, PDB 5HXE) and 2Na^+^ ions (**gray**, PDB 5HWX) are superimposed and viewed from the membrane plane (**left**) and from the extracellular side (**right**). Note the change in distance between TM6 and TM7ab induced by ligand binding; (**B**) the proposed “one-transition/two-occluded state” model for Ca^2+^ translocation from the extracellular to the cytosolic side, whereas the Ca^2+^-occluded state is more stable in the extracellular orientation (the outward-facing state) as compared with the cytosolic one (the inward-facing state). [E·Ca]^≠^ is the Ca^2+^-bound transition state; and (**C**) the Na^+^/Ca^2+^ exchange cycle is described as a separate translocation of 1Ca^2+^ or 3Na^+^, where the extracellular occlusion of either ion is more stable than the cytosolic one. **Green** and **red** spheres represent Na^+^ and Ca^2+^ ions, respectively. The gating bundle (TM1/TM6) is represented as a dashed line. In the frame of the “one-transition/two-occluded state” model, the movement of the TM1/TM6 bundle is driven by the transition state and not by the occlusion or semi-open states.

As a typical transporter, NCX conforms to the alternative excess dogma, according to which ligand binding domains are alternatingly exposed at opposite sides of the membrane, where adopting at least two major conformations, assigned as the inward-facing (IF) and outward-facing (OF) states [9,10,11,12,13,14]. In agreement with this, crystallographic studies identified the OF, IF, and occluded states in many transporter systems [12,13,14]. However, the conformational dynamics at specific elementary steps involved and the contributions of relevant conformational changes to functional undertakings remain largely unknown, even for transporters with a known crystal structure [9,10,11]. The recent achievements in the NCX field, summarized here, allow the description of the transport cycle with ground-state intermediates including the semi-open and occluded states (Figure 4). It is tempting to posit that ion-coupled alternating access in NCX proteins encompasses the “one-transition/two-occluded” state model with semi-open, occluded and transition state intermediates (Figure 4B,C). This model fundamentally differs from the often-used general models describing a central (single) occluded state, which is placed between the open (or semi-open) OF and IF states. The “one-transition/two-occluded” state model is especially interesting in light of a general postulate suggesting that ligand binding to the protein is obligatory for OF/IF swapping in antiporter systems, like NCX (i.e., in contrast with uniporters) [10,11]. By definition, only the transition state (and not the occluded and/or semi-open ground states) is capable of initiating the movement of the TM1/TM6 gating bundle toward OF/IF swapping (Figure 4B,C). Even though no experimental approaches/techniques are currently available for direct inspection of the transition state [7,8], a rational combination of X-ray crystallography, HDX-MS, kinetic analysis of mutants and MD simulations may lead to meaningful conclusions that may help in defining concrete objectives for resolving future challenges [24,25,26,27]. We describe below successful multidisciplinary approaches applied to the NCX_Mj protein, which can be explored for other transporters as well.

The HDX-MS analysis of NCX_Mj in the presence and absence of ligands provided insights into the mechanism underlying ligand-induced alternating access. Na^+^ and Ca^2+^ in saturating concentrations induced similar changes in deuterium uptake at the ion binding sites (Figure 3B–D), consistent with the notion that Na^+^ occupies S_Ca_ rather than S_mid_ [25,26,27]. In addition, the reduction of deuterium uptake at the extracellular portions of the α-repeats, upon ion binding, possibly reflects reduced solvent accessibility to an ion-permeation passageway with preference to adopt the ion-occluded state. Notably, the X-ray and HDX-MS data, in combination with MD simulations, strongly support the notion that the mechanisms underlying Na^+^ and Ca^2+^ occlusion might be comparable [25,26,27]. Note that deuterium uptake remains significant even under conditions of saturating ligand concentrations (Figure 3C,D), suggesting that although the occluded state becomes more populated, different states can be accommodated in the presence of either ligand. These findings are consistent with the notion that small structure-dynamic changes upon ion binding involve multiple local unfolding/refolding events with numerous low activation energies rather than a few large, concerted conformational changes, requiring high activation energies [26].

The HDX-MS measurements [26] in conjunction with known crystal structures and MD simulations [24,25,27] provided indispensable information on local conformational dynamics in the apo and ion-bound species of NCX_Mj. These HDX-MS data demonstrated that apo NCX_Mj exhibits hallmark patterns in the local backbone dynamics all along the TM2, TM3, and TM8 helices, thereby possessing signature profiles for structure-dynamic preorganization of the conserved α_1_ and α_2_ repeats and ion-coordinating residues within the ion-binding pocket [26]. The key feature of this signature preorganization is that TM2a and TM2b are much more constrained (less flexible) than are the other structural entities involved in ion transport events (TM2c, TM7a, TM7b, TM7c, TM8a, and TM8b). For example, local backbone dynamics of apo NCX_Mj are much more constrained at S51 and E54 (TM2c) than at T209 (TM7b), E213 (TM7c), and D240 (TM8a). Thus, conformational preorganization of specific local entities in the ligand-free protein might play a critical role toward stabilization of ion-bound species involved in the semi-open, occluded and transition states (see below) [24,26,27]. Moreover, the observed HDX kinetics revealed that both the apo and ion-bound species undergo multiple local unfolding/refolding steps, compatible with low activation energies rather than with a few large, concerted conformational changes, requiring high activation energies. This conclusion is consistent with mutational analyses of ion-flux activities, revealing small contributions (∆∆G) of key ion-coordinating residues to the stabilization of ion-bound species involved in ion transport catalysis [26]. In light of the present considerations, it is tempting to posit that structurally encoded preorganization of local structural entities at respective sites of the apo protein predefine the ion-occlusion and transition state features in NCX_Mj. The significance of this conceptual statement is that it underscores a critical role of structure-dynamic state(s) possessed by apo NCX_Mj, which actually predefine ion transport mechanisms, even though Na^+^ or Ca^2+^ binding moderately (but specifically) modifies the preexisting backbone dynamics nearby the key ion coordinating residues.

Recently, the extracellular-facing state of NCX_Mj was resolved in various occupancies of Ca^2+^, Na^+^ and Sr^2+^ bound species, thereby providing important information regarding the semi-open and occluded states of ion-bound species at the extracellular side [27]. Notably, the position of the gating bundle (the TM1/TM6 cluster) does not undergo any considerable conformational changes upon ion occlusion (Figure 4), thereby supporting a notion that the observed ion-bound species pave the way toward the transition state with resultant alternating-access transition. Namely, at low Na^+^ concentrations (<20 mM), only S_int_ and S_Ca_ are occupied, whereas higher concentrations of Na^+^ (up to 160 mM) are required for occupation of S_out_ [27]. The fully Na^+^-occupied exchanger adopts a conformation that matches the first reported crystal structure [24] and represents the outward-facing (extracellular) occluded state. Strikingly enough, the binding of the 3rd Na^+^ to S_ext_ bends the N-terminal halves of TM7 to two short helices, TM7a and TM7b, where TM7b occludes the ion-binding pocket from the extracellular solution. Moreover, at lower Na^+^ concentrations, the S_ext_ site is empty and TM7a and TM7b form a single straight helix resulting in a partially open state in which S_ext_ and S_mid_ are accessible. This bending of TM7 has an additional important outcome in forming a hydrophobic interface with the C-terminus of TM6, which may “precondition” the ion-coupled alternating access. Importantly, these contacts are absent in the presence of Na^+^ at S_ext_ (Figure 4). The Na^+^-dependent interaction of TM7 and TM6 is important, since TM1 and TM6 were hypothesized to slide against the rigid eight-helices protein core (TM2-TM5 and TM7-TM10) during alternating access, not only in NCX_Mj, but also in the three different crystal structures of the CAX proteins [24,27,32,33,34]. Therefore, the TM6-TM7 interactions may play a general and important role in decreasing the energy barrier for TM1/TM6 sliding. Although the inward-facing and outward-facing occluded states pave the way toward achieving a transition state, it is essential to affirm that the transition state (and not the occluded state either at the cytosolic or extracellular side) controls the ion-coupled movement of the gating bundle (TM1/TM6) in accomplishing the alternating-access transition [26]. The structural evidence for this claim is that, upon 3Na^+^ occlusion from the extracellular side, the gaiting bundle does not change its position [27]. This mechanism fundamentally differs from the common belief, suggesting a central (single) occluded state approaching the open (or semi-open) inward-facing and outward-facing states.

The structural evidence reveals that, in the presence of high Ca^2+^ concentrations, Ca^2+^ binds to both S_mid_ and S_Ca_, with a clear preference for S_Ca_. The binding to S_mid_ probably involves deprotonated D240; however, as mentioned above, D240 is protonated during ion transport, and, in most NCX orthologs, this position is occupied by an asparagine residue rather than aspartate. The Ca^2+^-bound exchanger adopts the partially open outward-facing conformation, similar to the 2Na^+^-bound state [24,27]. In the absence of ions, the free energy landscape allows only a fully open state. Upon 2Na^+^ binding, the partially open state is most favorable, but the fully open state is also energetically feasible [25,27]. Upon 3Na^+^ binding, the occluded state is most favorable, although at a relatively small energetic cost, the partially open state can also be occupied. Thus, sequential Na^+^ binding progressively reshapes the free-energy landscape of NCX, thereby leading to conformation transitions and presumably underlying alternating access. The Ca^2+^-bound state can accommodate the open, partially open, and occluded states; this is essential to allow Ca^2+^ binding/release (open), Ca^2+^ transport (occluded), and thus also the conformation in between (partially open). This is again congruent with the HDX-MS analysis suggesting that different conformations can be accommodated in the presence of either ligand [26].

## 7. Ion Selectivity in NCX Proteins and the Ca^2+^/Cation Antiporter Superfamily

**Figure 5:**
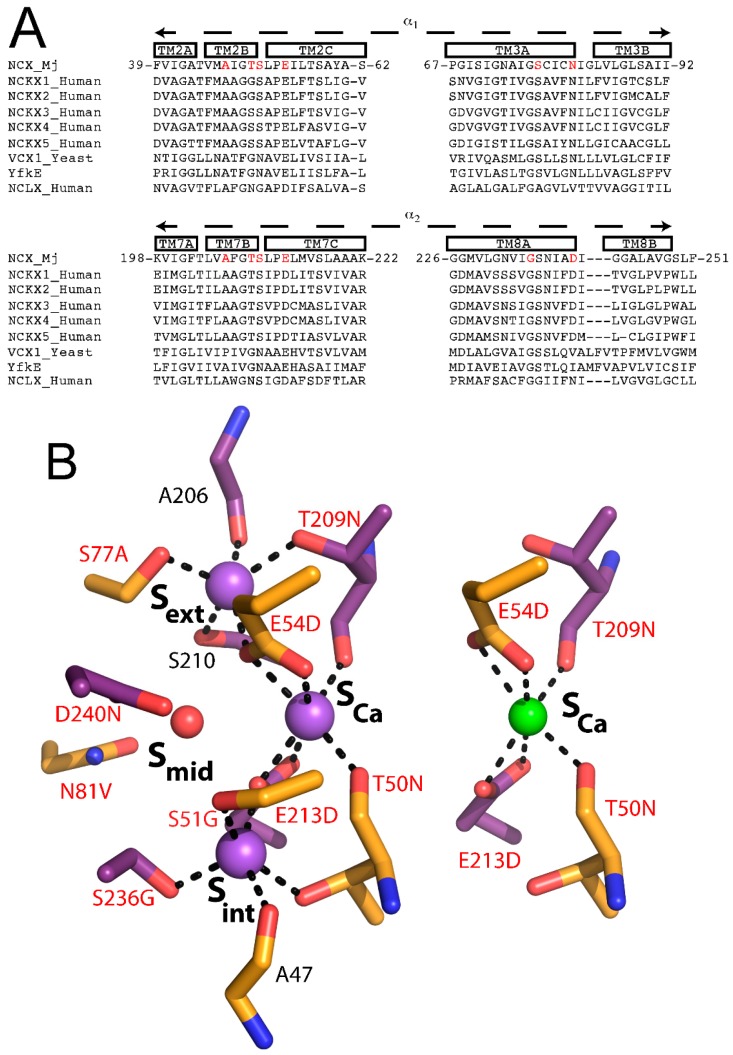
Ion selectivity of NCX. (**A**) sequence alignment of the α-repeats of NCX_Mj and members of the Na^+^/Ca^2+^-K^+^ exchanger (NCKX), Ca^2+^/H^+^ exchanger (CAX) and mitochondrial Na^+^/Ca^2+^ exchanger (NCLX) families. Ion-coordinating residues are **red**; (**B**) 3Na^+^ ions (**purple** spheres, 5HXE) coordination and 1Ca^2+^ ion (**green** sphere, 5HXR) coordination as suggested by the crystal structures of NCX_Mj. Mutated residues in the NCX_Mj/NCLX construct are **red**.

NCX proteins are members of the Ca^2+^/CA (Ca^2+^/cation antiporter) superfamily, which play an essential role in maintaining and modifying the cellular Ca^2+^ homeostasis in many cell types. All members of the Ca^2+^/CA exchangers superfamily facilitate the coupled transport of Ca^2+^ against its electrochemical gradient while transporting different cations, such as Na^+^ (NCX), K^+^ (NCKX), Li^+^ (NCLX), or H^+^ (CAX) along their electrochemical gradient in exchange with Ca^2+^ [16,17,32,33,34,40,41,42]. Despite their differences in ion selectivity, all members of the Ca^2+^/CA superfamily are predicted to share structural similarities with the α-repeats mediating the ion binding/transport events (Figure 5A). Moreover, based on the crystallographic structures of the H^+^/Ca^2+^ exchangers [32,33,34], all family members are expected to share a similar mechanism for alternating access involving the ion-induced movement of the gating bundle (TM1/TM6) (see above).

In attempt to decipher the structure–function relationships underlying ion selectivity in the Ca^2+^/CA superfamily, it was hypothesized that differences in the ion-coordinating residues underlie differences in ion selectivity among members of the Ca^2+^/CA superfamily [42]. To test this hypothesis, the Khananshvili laboratory has performed structure-based replacement of ion coordinating residues in NCX_Mj with residues from human NCLX (Figure 5B). More specifically, nine out of twelve ion coordinating residues were replaced simultaneously and the resultant mutant was tested for its capacity to mediate Na^+^/Ca^2+^, Li^+^/Ca^2+^ and Ca^2+^/Ca^2+^ exchange activities. Strikingly, this manipulation resulted in a novel capacity of the mutated NCX_Mj to transport either Na^+^ or Li^+^ in exchange with Ca^2+^, thereby suggesting that NCLX and NCX proteins share very common ion-transport mechanisms, where the ion-coupled alternating-access mechanism could be general as well [42]. Interestingly, the mutational effects on the turnover rates (*k*_cat_) were minor, whereas the effect on the K_m_ value for Na^+^ was reduced nearly 10-fold. Currently, it remains unclear whether the stoichiometry of ion-exchange is altered (e.g., only 2Na^+^ ions bind to S_int_ and S_Ca_), or, alternatively, the differences in the observed K_m_ values represent changes in the K_d_ values for 3Na^+^ binding affinity (Figure 3A). This specific example emphasizes the utility of the ion-flux assays in segregating the partial contributions of ion binding and ion transport in determining ion selectivity. Collectively, the structure-based design of Li^+^-transporting NCX_Mj demonstrates the potential use of NCX_Mj as a model system for studying the mechanisms underlying ion selectivity and alternating access in the Ca^2+^/CA proteins. An encouraging possibility from a biotechnological standpoint is that NCX_Mj may be used as a template for engineering novel transporters with predefined ion selectivity.

## 8. Conclusions

In recent years, the application of structural biology (X-ray crystallography, HDX-MS), computer-aided calculations (molecular dynamics), and biochemical assays (ion-binding and ion-flux monitoring, among others) have shed light on the structure–functional relationships underlying the ion-transport mechanisms in NCX proteins. The structural and functional data gained so far have provided a conceptual framework for studying the dynamic mechanisms by using the template model system of the archaeal NCX_Mj protein. Namely, the apo NCX_Mj protein exhibits hallmark patterns in the local backbone dynamics at specific segments of distinct helices and thereby possesses signature profiles for structure-dynamic preorganization of conserved α_1_ and α_2_ repeats at ion-coordinating residues involved in the ion-transport activities. In light of the present considerations, it is tempting to posit that specific preorganization of local structural entities in the apo protein predefines the features of ion-occlusion and transition states, even though Na^+^ or Ca^2+^ binding moderately (but specifically) modifies the preexisting backbone dynamics nearby the key ion coordinating residues. Future challenges include resolving the structural-dynamic determinants governing the ion selectivity and ion-induced alternating access. Taking into account the structural similarities of NCX_Mj with the other proteins of the Ca^2+^/CA superfamily, the recent findings can significantly contribute to a better understanding of ion-transport mechanisms in NCX and similar proteins.
